# Association of Apgar score at five minutes with long-term neurologic disability and cognitive function in a prevalence study of Danish conscripts

**DOI:** 10.1186/1471-2393-9-14

**Published:** 2009-04-02

**Authors:** Vera Ehrenstein, Lars Pedersen, Miriam Grijota, Gunnar Lauge Nielsen, Kenneth J Rothman, Henrik Toft Sørensen

**Affiliations:** 1 Department of Clinical Epidemiology, Aarhus University Hospital, Science Centre Skejby, Olof Palmes Allé 43-45, 8200 Aarhus N, Denmark; 2 Department of Epidemiology, Boston University School of Public Health, 715 Albany Street, Boston, MA 02118, USA; 3 RTI Health Solutions, Research Triangle Park, NC 27709, USA

## Abstract

**Background:**

Apgar score is used for rapid assessment of newborns. Low five-minute Apgar score has been associated with increased risk of severe neurologic outcome, but data on milder outcomes, particularly in the long term, are limited. We aimed to examine the association of five-minute Apgar score with prevalence of neurologic disability and with cognitive function in early adulthood.

**Methods:**

We conducted a prevalence study among draft-liable men born in Denmark in 1978–1983 and presenting for the mandatory army evaluation in a northern Danish conscription district. We linked records of this evaluation, which includes medical exam and intelligence testing, with the conscripts' records in the Medical Birth Registry, containing perinatal data. We examined prevalence of neurologic disability and of low cognitive function according to five-minute Apgar score.

**Results:**

Less than 1% (136/19,559) of the conscripts had 5-minute Apgar scores <7. Prevalence of neurologic disability was 2.2% (435/19,559) overall; among conscripts with Apgar scores <7, 7–9, and 10 (reference), it was 8.8%, 2.5%, and 2.2% respectively. The corresponding prevalences of low cognitive function (intelligence test score in the bottom quartile) were 34.9%, 27.2%, and 25.0%. The outcomes were more prevalent if Apgar score <7 was accompanied by certain fetal or obstetric adversities. After accounting for perinatal characteristics, 5-mintue Apgar score <7 was associated with prevalence ratios of 4.02 (95% confidence interval: 2.24; 7.24) for neurologic disability and 1.33 (0.94; 1.88) for low cognitive function.

**Conclusion:**

A five-minute Apgar score <7 has a consistent association with prevalence of neurologic disability and with low cognitive function in early adulthood.

## Background

Apgar score [1], used to evaluate infant's condition immediately after birth, is a sum of ratings (0, 1, or 2) of five clinical signs: heart rate, respiration, reflex irritability, muscle tone, and color. Five-minute Apgar scores below 4 are strong predictors of neonatal mortality [2]. Antenatal [3,4] and peripartum [5] adversities associated with five-minute Apgar scores below 7 [6-8] have been implicated in neonatal brain injury, which in turn may lead to neurodevelopmental disability [9-13].

Most newborns with Apgar scores below 7 grow up healthy, but risks of neurodevelopmental disability among them are greater than among those with higher Apgar scores, particularly in the short term [8,14-16]. Increased risks have been reported for neonatal seizures [8]; neonatal intracranial hemorrhage [8]; cerebral palsy [8,14,15]; mental retardation [8]; and epilepsy [16,17]. There are also reports of association between five-minute Apgar scores below 7 and risk of motor and developmental impairments at school age, including symptoms of attention deficit [18] and speech and language problems [19]. Less is known about long-term or mild neurodevelopmental disability among newborns with low Apgar scores. Recently, Odd et al., in a large study of Swedish conscripts, reported that worse cognitive performance was related to taking a longer time to achieve an Apgar score >6 [20]. This finding was in contrast to an earlier study by Seidman et al., who found little association between Apgar score and cognitive function among Israeli draftees [21]. Neither of the studies examined outcomes in men who are exempt from the draft. We studied the association of five-minute Apgar score with neurodevelopmental outcomes among Danish draftees. In addition to cognitive function, we examined prevalence of neurologic diseases, some of which are grounds for exemption from the military service.

## Methods

### Study population

We conducted a prevalence study among Danish men who were born as singletons in 1978–1983 and presented for conscription in 1996–2002. Nearly all men in Denmark are draft-liable and must register with authorities in one of the country's conscription districts. We studied men registered in the fifth conscription district, with jurisdiction primarily over the northern Danish counties of North Jutland and Viborg. During the registration, men report diseases that potentially preclude military service. Draft board physicians verify such reports with health care providers, and men with a verified condition receive exemption from the military duty. Among the remaining men, suitability for the service is determined in a routine evaluation, which includes a medical examination and intelligence testing [22]. Based on this evaluation, additional men may be deemed 'unfit' for the army. All disabilities reported to the draft board are recorded in the conscripts' files using Danish version of *International Classification of Diseases Tenth Revision *(ICD-10) [23]. We linked the conscription records with the corresponding records in the Danish Medical Birth Registry. The unambiguous individual-level linkage is enabled by a unique identifier that has been assigned to all Danes at birth since 1968 and has since been used in all administrative and health databases [24].

Our study population comprised men surviving to conscription age. Conscription districts are determined by place of residence at age 18 years, which may differ from place of birth. Thus, the exact birth cohort of boys destined for a given conscription district was unknown, and our study population is best thought of as a prevalence population rather than a cohort.

### Perinatal characteristics

The Danish Medical Birth Registry electronically tracks all births in Denmark since 1973 [25]. The data are entered from birth certificates filled out by midwives, who attend all births. From the Birth Registry, we extracted variables reportable at the time of the conscripts' birth: five-minute Apgar score, maternal age at delivery, marital status, parity; and newborn's birth weight, gestational age, total number of malformations, fetal presentation, and mode of delivery.

### Outcomes at conscription

We examined prevalent outcomes recorded at the time of conscription. We defined 'neurologic disability' as a record of a 'disease of the nervous system' in the conscription file (ICD-10 diagnoses G [23]). We defined 'disqualifying neurologic disability' as having one of the above diagnoses combined with being deemed 'unfit' for the military. Cognitive function was measured by the Boerge Prien test (Danish Børge Prien Prøve, BPP), which has been used for conscription purposes since 1957. It is a 78-item group intelligence test with four subscales (letter matrices, verbal analogies, number sequences, and geometric figures) and a single final score, recorded as the number of correctly answered items (range 0–78) [26]. BPP scores are strongly correlated with conventional intelligence-test scores (e.g., correlation of 0.82 with the Wechsler Adult Intelligence Scale [27]). For comparison with other studies, we converted Boerge Prien scores to the more conventional intelligence quotient (IQ) scale (mean = 100, standard deviation = 15 [28]) and examined distributions of the converted BPP scores and prevalence of low cognitive function, which we defined as a score in the bottom quartile.

### Data analysis

We examined all study outcomes according to five-minute Apgar score in categories <7, 7–9, and 10 (reference) [16]. We repeated these analyses in groups of maternal age at delivery (≤ 20, 21–35, >35 years); maternal marital status (married/unmarried); parity (0, ≥ 1); breech presentation; mode of delivery (vaginal, Cesarean, instrument); gestational age (<37, 37–41, ≥ 42 weeks) and birth weight small for gestational age (SGA), defined as weight <10^th ^percentile of all male live births in a given gestational week. We examined whether any association of Apgar score with the study outcomes could be explained by characteristics that are risk factors for both low Apgar score and for neurodevelopmental disability [9-13]. We used Zou's method of modified Poisson regression with robust error variance [29] to estimate prevalence ratios for neurologic disability and for low cognitive function; we used linear regression to estimate mean differences in IQ scores. Gestational age was missing for 17% of the conscripts owing to incomplete reporting in the earlier years of birth registration. To avoid loss of observations, we filled in missing values for gestational age using multiple imputation. The regression model used for imputation included variables for maternal age, marital status, parity, mode of delivery, conscript's birth year, birth presentation, birth weight, Apgar score at 1 minute, Apgar score at 5 minutes, neurologic disability, BPP score, and hearing and visual function measured at conscription [30]. Using a two-stage imputation procedure [31], we created five imputed datasets and averaged the estimates of effect for each outcome across the five datasets. The confidence intervals around these estimates reflect random error from the observed data and the uncertainty from the imputed values. We used SAS software, version 9.1 (SAS Inc., Cary, NC).

The study was approved by the Danish Registry Board. An informed consent was not required for this study of routine records.

## Results

### Descriptive data

Of the 19,843 draft-liable men born in 1978–1983 and registered with the fifth conscription district, 284 (1.4%) had missing data on five-minute Apgar score and were excluded. Of the remaining 19,559 men, 2336 (12%) men received health-related exemption before the formal evaluation, and therefore they did not undergo intelligence testing. The median age at the evaluation was 19 years (quartiles, 19–20 years). A large majority (93.4%) of the men had Apgar score of 10 at 5 minutes of age; 136 (0.7%) had Apgar score below 7, and 1143 (5.9%), a score in the range from 7 to 9. Five-minute Apgar score was inversely associated with prevalence of maternal nulliparity and unmarried status; and with prevalence of preterm birth, low birth weight, and Cesarean or instrument delivery (table 1).

**Table 1:** Perinatal characteristics of the 19 559 conscripts according to five-minute Apgar score

	**Five-minute Apgar score**			
		
	**<7**	**7–9**	**10**	**Total**
Number	136 (0.7%)	1143 (5.8%)	18 280 (93.5%)	19 559 (100.0%)
Mother's marital status				
Married	78 (57.4%)	727 (63.6%)	12 332 (67.5%)	13 137 (67.2%)
Unmarried	58 (42.6%)	416 (36.4%)	5948 (32.5%)	6422 (32.8%)
Parity				
0	82 (60.3%)	570 (49.9%)	7789 (42.6%)	8441 (43.1%)
1	29 (21.3%)	375 (32.8%)	6999 (38.3%)	7403 (37.9%)
≥ 2	25 (18.4%)	198 (17.3%)	3492 (19.1%)	3715 (19.0%)
Mother's age, years				
≤ 20 years	14 (10.3%)	119 (10.4%)	1724 (9.4%)	1857 (9.5%)
21–35 years	113 (83.1%)	956 (85.1%)	15 529 (85.9%)	16 598 (86.5%)
>35	9 (6.6%)	68 (5.9%)	1027 (5.6%)	1104 (5.6%)
Gestational age, weeks				
<37	28 (20.6%)	132 (11.6%)	543 (3.0%)	703 (3.6%)
37–42	74 (54.4%)	739 (64.6%)	13 260 (72.5%)	14 073 (71.9%)
≥ 42	14 (10.3%)	107 (9.4%)	1338 (7.3%)	1459 (7.5%)
Missing	20 (14.7%)	165 (14.4%)	3139 (17.2%)	3324 (17.0%)
Birth weight, gram				
<2500	41 (30.2%)	128 (11.2%)	553 (3.0%)	722 (3.7%)
≥ 2500	95 (69.8%)	1002 (87.6%)	17 598 (96.3%)	18 695 (95.6%)
Missing	0 (0.0%)	13 (1.1%)	129 (0.7%)	142 (0.7%)
SGA	18 (13.2%)	117 (10.2%)	1473 (8.1%)	1608 (8.2%)
Mode of delivery				
Vaginal unassisted	69 (50.7%)	681 (59.6%)	14 895 (81.5%)	15 645 (80.0%)
Cesarean delivery	34 (25.0%)	234 (20.5%)	1728 (9.4%)	1996 (10.2%)
Instrument delivery	33 (24.2%)	228 (19.9%)	1657 (9.1%)	1918 (9.8%)
Fetal presentation				
Cephalic	111 (81.6%)	1026 (89.8%)	17 183 (94%)	18 320 (93.7%)
Breech	23 (16.9%)	105 (9.2%)	903 (4.9%)	1031 (5.3%)
Missing	2 (1.5%)	12 (1.0%)	194 (1.1%)	208 (1.1%)

### Neurologic disability

A neurologic disability as defined here was reported at conscription for 435/19,559 (2.2%) of the men (table 2). The prevalence of neurologic disability among men with a five-minute Apgar score <7 was 8.8% (12/136), and it was 2.5% (29/1143) among men with five-minute Apgar scores in the 7–9 range. A disqualifying neurologic disability was recorded among 273 (1.4%) of the conscripts, with epilepsy (ICD-10 codes G40.x) and cerebral palsy (ICD-10 codes G80.x-G83.x) accounting for 60% of the diagnoses. Among the 168 conscripts with neurologic disability who were not considered unfit, the most frequent diagnosis was migraine (73%). Among the 435 men with a neurologic disability, the proportion of disqualified men was inversely related to the Apgar score: 11/12 (92%; 95% CI: 61% – 100%); 20/29 (68%; 95% CI: 49% – 85%), and 242/394 (61%; 95% CI: 56% – 66%) in five-minute Apgar scores categories <7, 7–9, and 10, respectively.

**Table 2:** Neurologic disability and low cognitive function according to five-minute Apgar score.

	**All conscripts**			**Conscripts with measured cognitive function**	
		
**Five-minute Apgar score**		**Neurologic disability^a^**	**Disqualifying neurologic disability^b^**	**Low cognitive function^c^**	
					
	**N**	**n (%)**	**n (%)**	**N**	**n (%)**
					
<7	136	12 (8.8)	11 (8.1)	106	37 (34.9)
7–9	1143	29 (2.5)	20 (1.8)	992	244 (27.2)
10	18 280	394 (2.2)	242 (1.3)	16 113	3562 (25.0)
					
Overall	19 559	435 (2.2)	273 (1.4)	17 211	4344 (25.2)

^a ^Defined as a record of a diagnosis G ('diseases of nervous system' in ICD-10), noted in conscripts' draft board record.

^b ^Twenty-one men had no entry for army fitness.

^c ^Defined as an IQ in the bottom quartile

### Cognitive function

**Figure 1:**
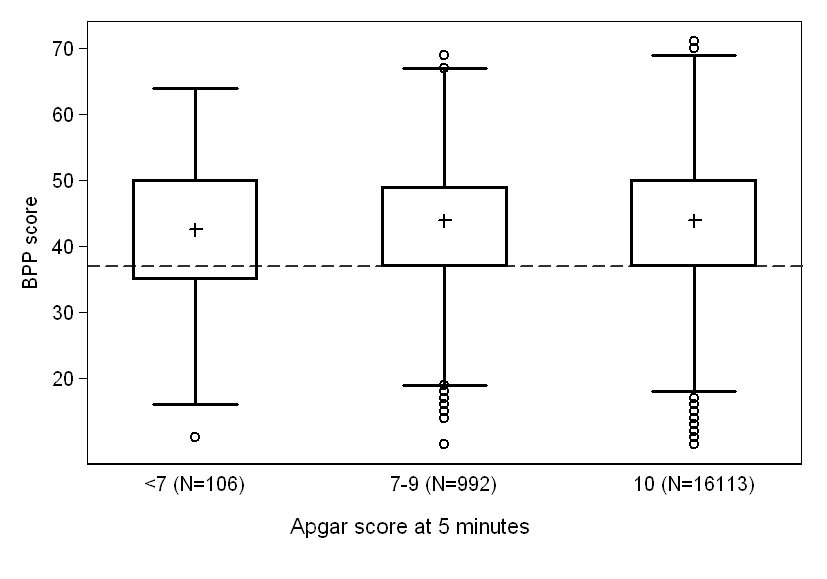
**Box-and-whisker plots of Boerge Prien test scores converted to the conventional IQ scale (mean = 100, standard deviation = 15) according to Apgar score at 5 minutes**. Pluses indicate median values; dashed line marks the bottom quartile of the overall distribution.

Among the 17,223 men who underwent the intelligence testing, we excluded eight records with a missing score and additional four records with BPP scores below 10, all of which we considered data entry errors. Among the remaining 17,211 men, IQ scores were normally distributed, and the distributions did not differ substantially according the Apgar score categories (figure 1). Compared with those whose five-minute Apgar score was 10, mean differences in IQ scores were -2.6 points (95% CI, -5.4; 0.3) and -1.0 points (95% CI, -1.9; 0.0) for men with 5-mintue Apgar scores <7 and 7–9, respectively. Prevalence of low cognitive function among those with a five-minute Apgar score <7 was 34.9%, which was greater than the 25% expected given our definition of low cognitive function as a score in the bottom quartile (table 2). The observed prevalence of low cognitive function differed from the expected value of 25% among conscripts who were born to mothers aged 20 years or younger (36.3%) or those who were SGA (32.9%). Greater differences from the expected distribution were seen among those who had five-minute Apgar score <7 coupled with maternal age 20 years or younger (60.0%); with preterm birth (40.9%); with SGA (50.0%); or with instrument delivery (48.1%). These estimates are based on small number of 'events'.

### Regression modeling

Additional file 1 shows results of regression modeling. Inclusion in the model of the measured maternal and fetal characteristics caused only slight attenuation in the crude prevalence ratios for Apgar scores <7 and 7–9. We excluded the variable for mode of delivery from the final model, as it had no effect on the estimates. After controlling for other covariates, five-minute Apgar score <7 was associated with a four-fold increase in the prevalence of neurologic disability (prevalence ratio = 4.02 (95% CI, 2.24; 7.24)), and with nearly a six-fold increase in prevalence of disqualifying neurologic disability (prevalence ratio = 5.94 (95% CI, 3.19; 11.06)). We saw a similar pattern for low cognitive function, but this measure is expected to be weaker because the prevalence in the reference group is by definition around 25%, limiting the maximum ratio to 4. For this measure we found a prevalence ratio of 1.33 (95% CI, 0.94; 1.88) after controlling for the covariates. The mean difference in conventional IQ score was -1.8 (95% CI, -4.7; 1.0) and -0.9 (95% CI, -1.9; 0.0), for five-minute Apgar score <7 and 7–9, respectively, compared with Apgar score of 10, after controlling for covariates. Similar estimates were obtained in complete-subject analyses (done without imputation) or after excluding men with a record of a malformation in the Birth Registry (N = 292, data not shown).

## Discussion

Having an Apgar score below 7 at five minutes was associated with greater prevalence of neurologic disability and of low cognitive function among Danish draftees. An Apgar score <7 may be a marker for severity of neurologic impairment, as suggested by its inverse association with proportion of disqualifying neurologic diagnoses. The absolute risk increases of 6.6% for neurologic disability and 9.9% for low cognitive function associated with five-minute Apgar score <7 imply a low sensitivity and thus a limited clinical utility in predicting long-term disability. At the same time, we found limited evidence of the absolute increase in risk of low cognitive function rising to 15%–35% if Apgar score <7 was accompanied by very young maternal age, growth restriction, or instrument delivery. Accounting for measured perinatal characteristics attenuated but did not fully account for the observed associations. The results indicate that low Apgar score is associated with impaired neurodevelopment through several mechanisms, only some of which involve clinical characteristics typically observed at birth. Five-minute Apgar scores in the 7–9 range were also associated with worse outcomes in our data, which is consistent with the notion of gradual increase in risk with worsening of condition at birth.

Our findings regarding Apgar score and neurologic disability are in agreement with recent reports of an inverse association between five-minute Apgar score and long-term risk of epilepsy [16,17]. Unlike those studies, which ascertained epilepsy from computerized hospitalization records, we used diagnoses reported directly to physicians, which should be less likely to include false-positive diagnoses [32]. Our findings regarding cognitive function corroborate and extend existing knowledge. Odd et al., in a population of >130,000 Swedish draftees born in early 1970s, found a small reduction in mean IQ scores associated with low Apgar scores, similar in magnitude to our findings. The estimate of risk ratio for poor cognitive function in our study, 1.33 (0.94–1.88), and that from the study of Odd et al., 1.35 (95% CI: 1.07–1.69) [20] were similar despite the latter study defining poor cognitive score as the bottom 9% of the distribution. In clinical practice, cutoffs of <1 and <2 standard deviations below the mean IQ are commonly used. In our data, prevalence ratios for those two definitions of low cognitive function associated with Apgar score <7 were, respectively, 1.44 (95% CI: 1.00–2.07) and 1.74 (95% CI: 0.80–3.81). These outcomes were not used in the main analyses because of small number of 'events' in the Apgar <7 group. Lawlor et al. found a 5-minute Apgar score <8 to be associated with a 1.6-point mean decrease in IQ among adolescents at age 14 years [33], a value similar to our estimate of a 1.8-point mean decrease. Seidman et al. found a nearly null association among Israeli conscripts [21] based on the examination of mean differences. We do not interpret a mean IQ decrease on the order of one-tenth of one standard deviation as being clinically significant. On the other hand, a small population shift could reflect a more important deficit for subgroups of a population. By analogy, small shifts in the mean blood pressure of a population may be important for specific subgroups [34]. Thus, examining only mean difference may mask effects seen only in the fringes of the total distribution.

We estimated potential loss to follow-up from the underlying birth cohort in a sample of 14,288 boys born in the study area in 1980–1983. Before reaching conscription age, the emigration out of Denmark was <1%; and mortality was 1.2%. For boys with five-minute Apgar scores below 7, mortality before age 1 year was 28%, most of the deaths occurring among boys with scores below four. The overall mortality before age 1 year was 0.8%, which is consistent with the period nationwide estimates [35]. We had no information on potentially eligible men who may have been institutionalized for legal or health reasons. Thus, at conscription age, men with a history of low Apgar score appearing before the draft board represent a comparatively healthy subset of all newborns with low Apgar scores. As noted by Odd et al., the differential survival underscores the fact that associations between neonatal condition and adult neurodevelopment seem to persist even in relatively healthy men [20].

Misclassification of the newborn condition by recorded Apgar score could result from random data entry errors and from the partially subjective nature of the Apgar score. These errors can be assumed to be independent of the outcomes we examined and therefore unlikely to cause upward bias in our measures of association. Neurologic diagnoses among conscripts may be under-ascertained by the draft-file information, if only the first-reported disqualifying diagnosis is recorded. Nevertheless, the prevalence of neurologic conditions in our sample (2.2%) is comparable with published population figures [36]. Accounting for measured perinatal characteristics caused prevalence ratios to decrease, implying that using better-measured or additional factors theoretically could reduce the observed associations. For example, accounting for socioeconomic markers other than maternal characteristics could explain part of the associations with cognitive function, as it did the Swedish cohort [20].

## Conclusion

In conclusion, we found that certain indicators of adult neurologic function and its severity are present at birth and are indirectly measurable even with a crude index such as the Apgar score.

## Competing interests

The authors declare that they have no competing interests.

## Authors' contributions

VE, HTS, GLN, and KJR all substantially contributed to study conception, design and interpretation of data. VE drafted the article and analyzed the data. LP, MG, and GLN participated in dataset creation and data analysis. All authors revised the paper critically and gave their approval of the final version. VE is the guarantor.

## Pre-publication history

The pre-publication history for this paper can be accessed here:



## Supplementary Material

Additional file 1 **Table**. Association of five-minute Apgar score with neurologic disability and cognitive function. Click here for file
