# Supplementary material for: Association of Apgar score at five minutes with long-term neurologic disability and cognitive function in a prevalence study of Danish conscripts
Source: BMC Pregnancy Childbirth. 2009 Apr 2;9:14. doi: 10.1186/1471-2393-9-14 (PMC2670812; doi:10.1186/1471-2393-9-14)
Supplement: Additional file 1 — Table. Association of five-minute Apgar score with neurologic disability and cognitive function. [file 1471-2393-9-14-S1.pdf]

Table 3: Association of five-minute Apgar score with neurologic disability and cognitive function.[a]

Apgar score at 5 minutes	Any neurologic disability		Disqualifying neurologic disability		Low cognitive function[b]	
	Crude	Accounting for perinatal characteristics[c]	Crude	Accounting for perinatal characteristics[c]	Crude	Accounting for perinatal characteristics[c]
<7 vs. 10	4.09 (2.36; 7.09)	4.02 (2.24; 7.24)	6.10 (3.41; 10.90)	5.94 (3.19; 11.06)	1.39 (1.07; 1.81)	1.33 (0.94; 1.88)
7-9 vs. 10	1.18 (0.81; 1.71)	1.13 (0.77; 1.67)	1.32 (0.84; 2.08)	1.31 (0.82; 2.07)	1.09 (0.98; 1.21)	1.08 (0.94; 1.23)

[a] Entries are prevalence ratios (95% confidence intervals).

[b] Measured for 17 211 men who underwent the draft board evaluation.

[c] Models include 5-minute Apgar score (<7, 7-9, 10); maternal age (≤20, 21-35, >35 years); marital status (married/unmarried); parity (0, ≥1); breech presentation; gestational age (<37, 37-41, ≥42 weeks) and small for gestational age (SGA, defined as birth weight <10 percentile of birth weight among male live births for each completed week of gestation).
